# Acute Cholecystitis in a Patient With Situs Inversus Totalis: An Unexpected Finding

**DOI:** 10.7759/cureus.15799

**Published:** 2021-06-21

**Authors:** Andres Felipe Herrera Ortiz, Juan C Lacouture, Daniel Sandoval Medina, Luis J Gómez Meléndez, Rodolfo Uscategui

**Affiliations:** 1 Radiology, Universidad El Bosque, Bogotá D.C., COL; 2 Surgery, Clínica del Country, Bogotá D.C., COL; 3 Surgery, Universidad del Rosario, Bogotá D.C., COL; 4 Surgery, Universidad Nacional, Bogotá D.C., COL

**Keywords:** cholecystectomy laparoscopic, situs inversus, cholecystitis, cholelithiasis, abdominal pain

## Abstract

Situs inversus totalis (SIT) has an incidence in the general population of 1/10,000, with a female-male ratio of 1:1.5 without racial predilection. Clinically, SIT by itself tends to be asymptomatic; however, when it is associated with other conditions such as cholecystitis or appendicitis, the diagnosis may represent a challenge due to the reversed anatomical location of symptoms.

This article presents a case of a 46-year-old female who arrived at the emergency department due to one week of non-bilious vomiting and colicky abdominal pain located in the left hypochondrium; therefore, abdominal ultrasonography was performed, showing transposition of abdominal organs associated with cholelithiasis plus acute cholecystitis. As a result, the patient was scheduled for laparoscopic cholecystectomy, resulting in an appropriate post-surgical evolution, for which discharge was given with a general surgery control appointment.

Laparoscopic cholecystectomy in patients with SIT represents a challenge due to the technical complexity derived from the transposition of the abdominal organs; therefore, the surgeon is forced to perform the procedure by placing three trocars with a specular approach plus the umbilical trocar.

## Introduction

Situs inversus totalis (SIT) has an incidence in the general population of 1/10,000, with a female-male relationship of 1:1.5, with no racial preference [1-3]. Anatomically SIT is presented as a total transposition of the abdominal and thoracic organs. Clinically, it tends to be asymptomatic; therefore, it is usually diagnosed when consulting for other diseases that demand imaging modalities [1,2].

The usual causes of acute abdominal pain in the general population can easily lead to misdiagnosis when the patient has SIT. For example, in the case of appendicitis, pain may be located on the left iliac fossa, leading to misdiagnosing it as acute diverticulitis. In cholecystitis, the pain could be found in the left upper quadrant, easily misdiagnosing it as gastritis. Therefore, the physician must keep clinical suspicion of SIT based on physical examination findings and subsequently perform the respective radiological studies [4-6].

This article seeks to describe and discuss the case of a patient diagnosed with SIT plus cholelithiasis and cholecystitis who underwent laparoscopic cholecystectomy. This case represents a surgical challenge due to the technical difficulty derived from the transposition of the abdominal organs.

## Case presentation

A 46-year-old female living in a rural area arrived at the emergency department due to one week of non-bilious vomiting and colicky abdominal pain located in the left hypochondrium, which increases with fatty food intake. The patient referred that her sister was diagnosed with SIT 10 years ago. On physical examination, a patient in stable general conditions was evident, hydrated, afebrile, with left Murphy's sign and normal heart sounds on the right side of the thorax.

**Figure 1:**
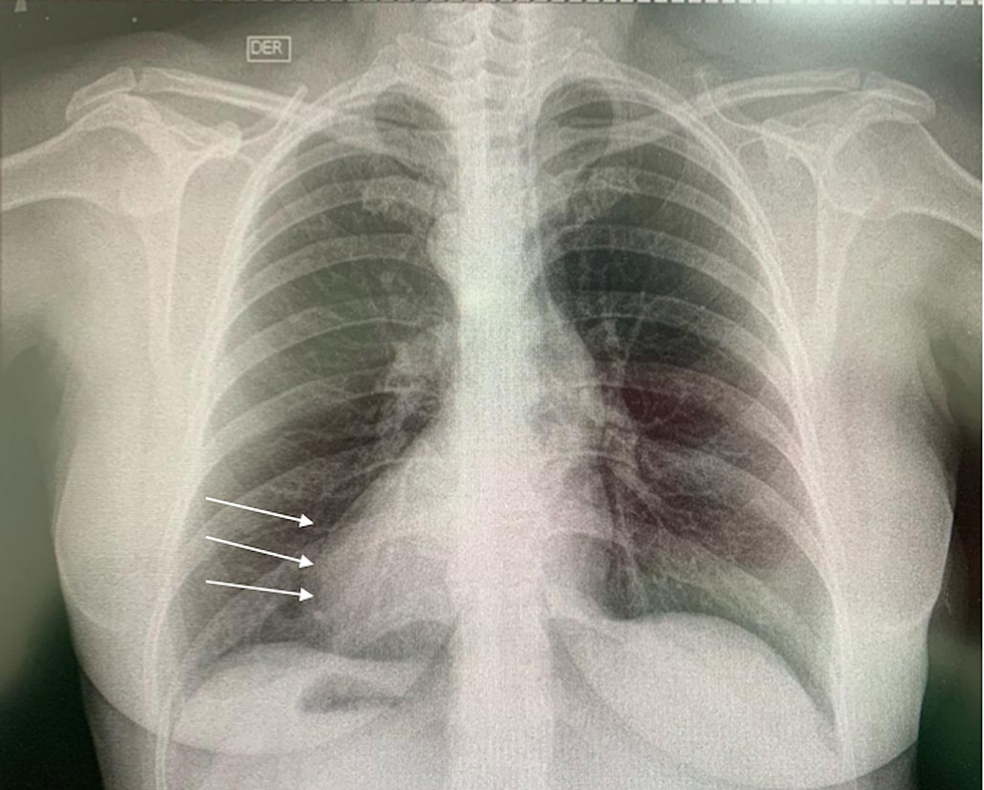
Chest radiography showing dextrocardia (white arrows)

Due to the physical examination findings and the family history of SIT, abdominal ultrasonography was performed, showing transposition of abdominal organs associated with cholelithiasis + acute cholecystitis (common bile duct measured 4.3 mm). Chest radiography was requested to assess the heart's position, showing dextrocardia with gastric bubble located to the right and liver situated to the left (Figure 1), confirming the diagnosis of SIT. The liver function tests were: alkaline phosphatase 102 UI/L, total bilirubin 2.2 mg/dl, direct bilirubin 2 mg/dl, alanine aminotransferase (ALT) 26 UI/L, aspartate aminotransferase (AST) 22 UI/L. An echocardiogram was performed to rule out SIT with cardiovascular compromise, revealing a heart located to the right side without other cardiovascular affection. The electrocardiogram showed an electric heart axis to the right.

**Figure 2:**
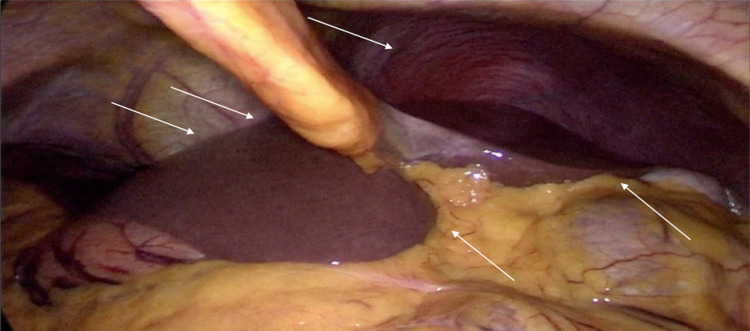
Laparoscopy showing transposition of abdominal organs. Liver located to the left side (white arrows).

Due to the diagnosis of cholelithiasis + cholecystitis with a low probability of choledocholithiasis, the patient was scheduled for laparoscopic cholecystectomy. A diagnostic laparoscopy was performed with the surgeon and the camera assistant located to the right, while the monitor and the first assistant situated to the left side of the patient, showing SIT with the liver to the left hypochondrium, cecal appendix in the left iliac fossa, and spleen in the right hypochondrium (Figure 2).

**Figure 3:**
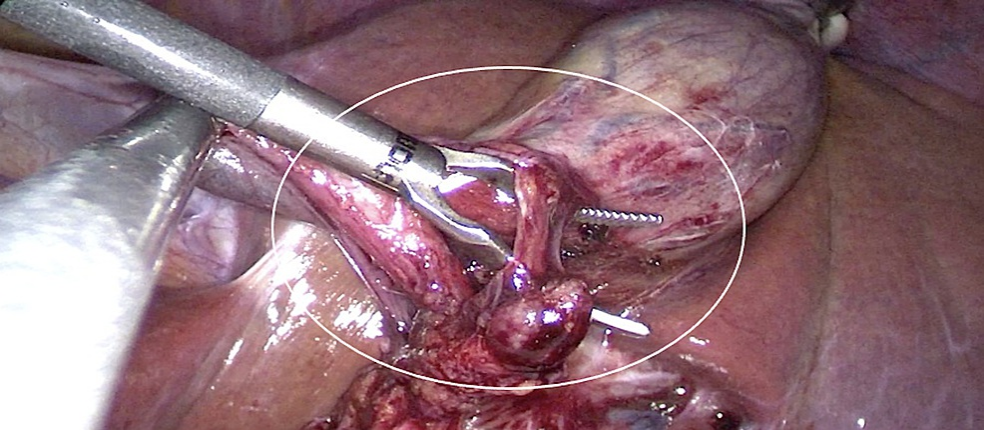
Laparoscopy. Dissection of the Calot triangle (white circle).

The surgical technique was the same as routine but with the particularity of putting the trocars in opposite positions, as if operating in a mirror. As usual, the dissection of the Calot's triangle was performed in Strasberg's safety window with the clipping of the cystic artery and the cystic duct, which were in their normal positions (Figure 3). The gallbladder was removed, showing cholelithiasis inside. The procedure was carried out without complications, showing a pathology report of cholelithiasis with cholecystitis.

After the procedure, the patient presented a favorable post-surgical evolution with tolerance to regular diet and improvement of abdominal pain; therefore, discharge was given with analgesia and general surgery control appointment. The patient was evaluated one week and one month after surgery showing good general condition and resolution of pain; no post-surgical complications were presented.

## Discussion

SIT is a rare anatomical variant; it is believed that it is caused by a modification in the lefty1 gene, PITX2, and fibroblast growth factor eight, affecting the right and left axis of the embryo during the gastrulation stage in the third week of gestation [3]. The inherited mechanism is debated; some authors suggest an autosomal recessive pattern, while others indicate an autosomal dominant mechanism [2,7]. It is essential to highlight that the patient and her sister had SIT while the parents did not; therefore, this presentation suggests an autosomal recessive inheritance pattern, as some authors have described previously [1,8,9].

The SIT by itself is asymptomatic; however, it may be accompanied by cardiovascular alterations such as atrial situs solitus (93%), discordant atrioventricular connection (44%), and discordant ventriculoatrial connection (30%); gastrointestinal and vascular alterations have also been reported, but its frequency is not well established in the literature [10-13]. SIT can also be associated with other entities such as interruption of the vena cava, Kartagener syndrome, Ivermark syndrome, and Yoshikawa syndrome [3,4,11]. Close to 25% of patients with dextrocardia will have Kartagener syndrome [5]. Patients with acute cholecystitis plus SIT present pain in the left upper quadrant or epigastric region in just 30% of cases [8]. On the other hand, only 50% of patients with left side appendix experience pain in the left side. This clinical presentation can be explained by the fact that the organs are transposed, but the peripheral nervous system components are not, allowing the patient to experience diffuse abdominal pain [6].

Currently, there is insufficient evidence to demonstrate an association between SIT and the appearance of gallstones, so its incidence is equal to the general population [2]. Some authors have described an increased incidence of gallbladder malformations in patients with SIT; therefore, preoperatory magnetic resonance imaging (MRI) can be helpful during surgery [14,15]. In this case, it was not performed due to the limited availability of MRI in our institution. In literature, there are reported cases of laparoscopic cholecystectomies in patients with SIT, which have been performed with success and a low rate of complications due to pre-surgical knowledge of the condition. All patients with SIT should be evaluated pre-surgically to rule out associated syndromes and cardiac or gastrointestinal malformations that may complicate the surgical act [4,6].

**Figure 4:**
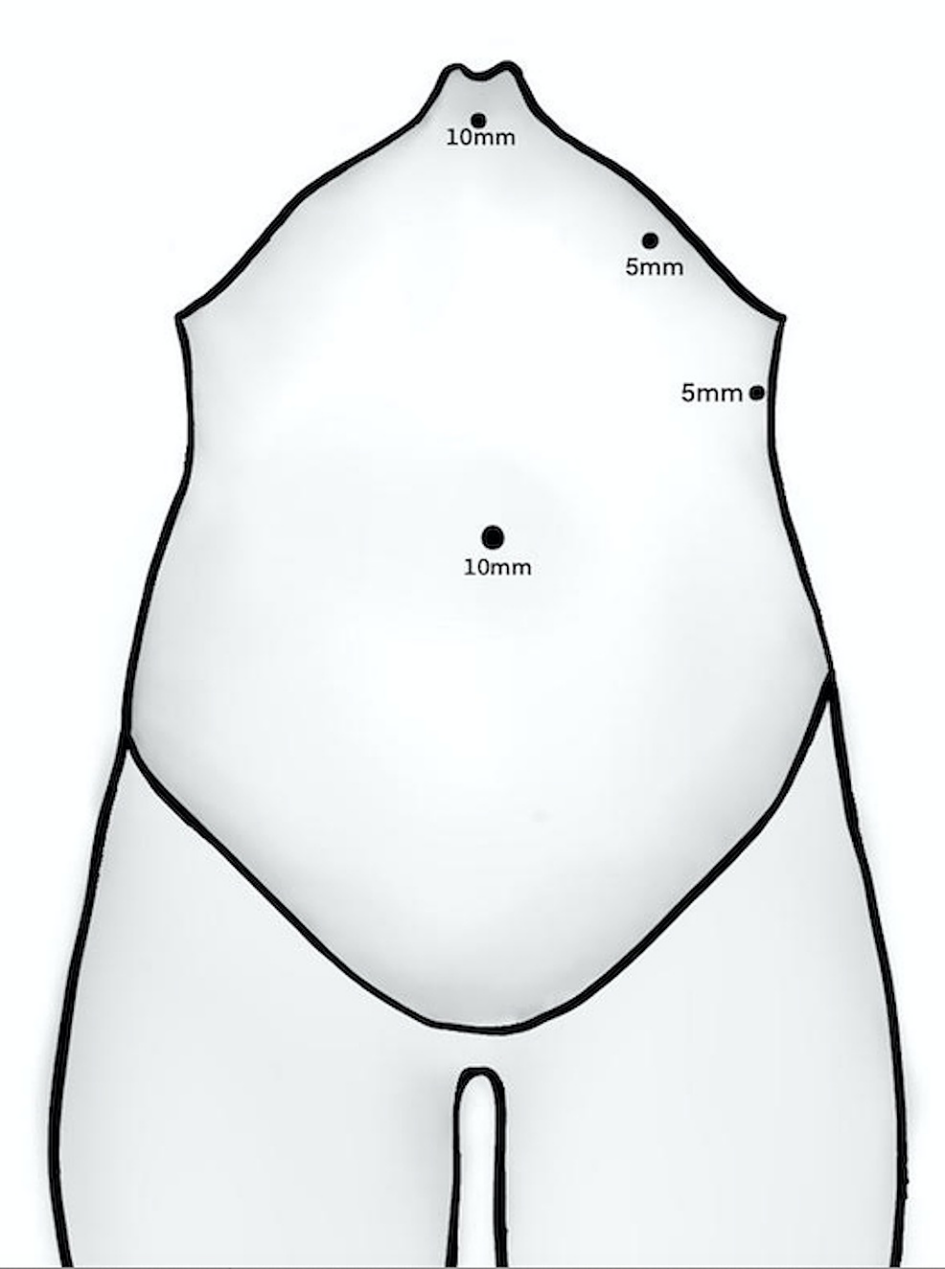
Position and size of the ports during specular laparoscopy Source: elaborated by the authors.

Surprisingly, our patient was not diagnosed with SIT until 46 years old, considering the fact that her sister had the diagnosis 10 years ago. We think that it can be explained due to the limited health care access in the rural population in our country. In this case, there was pre-surgical knowledge of the SIT by an in-hospital abdominal ultrasound, cardiac auscultation, and chest X-ray, thus facilitating the procedure given to proper surgical planning. Laparoscopic cholecystectomy in patients with SIT has been reported in several articles; the most widely used technique described in the literature is by placing three trocars with a specular approach plus the umbilical trocar (Figure 4), with the surgeon and the camera assistant located to the right, while the monitor and the first assistant situated to the left side of the patient. This approach facilitates the use of the left hand to hold the Hartmann’s pouch through the subxiphoid port and the right hand to perform the dissection through the left midclavicular subcostal ports [15]. Some authors have reported laparoscopic cholecystectomies in patients with SIT performed by right-handed surgeons located to the patient's left side, describing issues to fulfill the dissection due to conflicts between instruments; therefore, right-handed surgeons are expected to be positioned to the patient's right side, as was done in this case report [16].

The mirror appearance of the abdominal organs makes the surgery more challenging due to difficulties in spatial orientation and the detection of anatomical variants, leading to an increased surgical time [4,15]. Therefore, it could be said that a surgeon whose dominant hand is the left could have more advantage to perform a laparoscopic cholecystectomy in a patient with SIT [4,8]. A single port laparoscopic cholecystectomy approach has also been described in a few case reports, showing an improvement in the dissection of the vascular and biliary structures for the right-handed surgeon; nevertheless, the main disadvantage is that the surgeon requires to be constantly moving across the patient body leading in increased fatigue [15-17]. In contrast, other authors have suggested performing the surgery with the patient in Lloyd-Davis's position and the surgeon between the patients' legs [18,19].

## Conclusions

Finally, it is crucial to maintain a clinical suspicion of SIT in all patients with pain in the left upper quadrant since it is easy to confuse it with gastritis or other gastric condition. Therefore, all patients with SIT should be thoroughly evaluated preoperatively to rule out gastrointestinal and cardiac malformations as well as associated syndromes that may complicate the surgical act. The surgical approach chosen depends on the surgeon's preferences (either with one trocar, three trocars on specular localization plus the umbilical port, or the surgeon between the patients' legs); nevertheless, the procedure tends to be highly challenging due to the transposition of abdominal organs, increasing the surgical time of the intervention.
